# Hierarchically tumor-activated nanoCRISPR-Cas13a facilitates efficient microRNA disruption for multi-pathway-mediated tumor suppression

**DOI:** 10.7150/thno.81776

**Published:** 2023-05-08

**Authors:** Xiaowei Liu, Suleixin Yang, Li Wang, Xinyue Wu, Xinxin Wang, Chunqing Ou, Jin Yang, Linjiang Song, Shiyao Zhou, Qinjie Wu, Changyang Gong

**Affiliations:** 1 State Key Laboratory of Biotherapy and Cancer Center, West China Hospital, Sichuan University, Chengdu 610041, P. R. China.; 2 School of Medical and Life Sciences, Chengdu University of Traditional Chinese Medicine, Chengdu, 611137, P. R. China.

**Keywords:** tumor-activated, CRISPR-Cas13a, microRNA disruption, multi-pathway regulation, cancer treatment

## Abstract

**Rationale:** CRISPR-Cas13a is an efficient tool for robust RNA knockdown with lower off-target effect, which may be a potentially powerful and safe tool for cancer gene therapy. However, therapeutic effect of current cancer gene therapy that targeting monogene was compromised by the multi-mutational signal pathway alterations of tumorigenesis.

**Methods:** Here, hierarchically tumor-activated nanoCRISPR-Cas13a (CHAIN) is fabricated for multi-pathway-mediated tumor suppression by efficient microRNA disruption *in vivo*. A fluorinated polyetherimide (PEI; Mw=1.8KD) with graft rate of 33% (PF_33_) was utilized to compact the CRISPR-Cas13a megaplasmid targeting microRNA-21 (miR-21) (pCas13a-crRNA) via self-assemble to constitute a nanoscale 'core' (PF_33_/pCas13a-crRNA), which was further wrapped by modified hyaluronan (HA) derivatives (galactopyranoside-PEG2000-HA, GPH) to form CHAIN.

**Results:** The dual-tumor-targeting and tumor-activated CHAIN not only manifested long-term circulation, but augmented tumor cellular uptake and endo/lysosomal escape, thus achieving efficient transfection of CRISPR-Cas13a megaplasmid (~ 13 kb) in tumor cells with minimal toxity. Efficient knockdown of miR-21 by CHAIN restored programmed cell death protein 4 (PDCD4) and reversion‐inducing‐cysteine‐rich protein with Kazal motifs (RECK) and further crippled downstream matrix metalloproteinases-2 (MMP-2), which undermined cancer proliferation, migration and invasion. Meanwhile, the miR-21-PDCD4-AP-1 positive feedback loop further functioned as an enhanced force for anti-tumor activity.

**Conclusion:** Treatment with CHAIN in hepatocellular carcinoma mouse model achieved significant inhibition of miR-21 expression and rescued multi-pathway, which triggered substantial tumor growth suppression. By efficient CRISPR-Cas13a induced interference of one oncogenic microRNA, the CHAIN platform exerted promising capabilities in cancer treatment.

## Introduction

Along with the mounting knowledge of tumorigenesis molecular mechanisms, gene therapy offers a potential choice for cancer treatment 1,2. Current cancer gene therapy mainly focuses on delivering RNA/DNA system targeting one single gene 3-7. However, neoplastic cells are driven by a combination of gene abnormalities or multiple signal pathway alterations on the genetic level, which led to tumorigenesis 8-10. Due to the sophisticated mechanism of cancer, targeting only one signaling molecules in cancer has shown only short-lived or modest clinical benefit 11. Thus, it is becoming increasingly clear that cancer gene therapy should regulate several targets in the multi-pathway signal network.

MicroRNAs (miRNAs), one class of small noncoding RNAs, were found to interfere the gene expression and subsequently promote or inhibit cell differentiation, proliferation and apoptosis *via* regulating multiple downstream genes 12,13. The abnormal expression of miRNAs contributes to various cancers 14. Many solid cancers contain high levels of the oncogenic microRNA miR-21 (miR-21), whose overexpression encourages cancer cell invasion, metastasis, proliferation, and tumor formation 15-17. Therefore, inhibiting miR-21 expression and modulating the downstream multi-signal pathways may be an ideal strategy for cancer therapy.

The clustered regularly interspaced short palindromic repeat (CRISPR)/CRISPR-associated protein (Cas) adaptive immunity systems evolved to protect bacteria and archaea against foreign nucleic acids of viruses and other genetic elements 18-21. Among them, CRISPR-Cas13a (originating from *Leptotrichia wadei*), has been reported as an efficient RNA knockdown tool with competitive efficiency to RNA interference (RNAi) technology 22-24. Cas13a recognizes and cleaves single-stranded RNA target with a protospacer flanking sequence (PFS: A, U, or C) by the direction of a single CRISPR RNA (crRNA) containing a 28-nt spacer sequence, which largely minimized the off-target effects 22. Furthermore, due to its direct RNA targeting, CRISPR-Cas13a system can avoid potential risks induced by manipulating genomic DNA, such as chromosomal instability and oncogene activation 25,26, which showed great potential for cancer treatment by gene edting. However, the large size of CRISPR-Cas13a plasmid (~ 13 kb) still challenges its safe and effective delivery *in vivo*.

The exertion of genetic therapy system calls for sufficient editing system molecules inside target cells 6,27,28. Therefore, an efficient and non-cytotoxic delivery system will be the basic prerequisite for effective therapeutic application. Currently, due to their long-term expression, high infection efficiency and wide hosts variety, viral vectors are powerful weapons for gene therapy and oncolytic viral therapy 29-31. Most viruses are nanoscale particles with a 'core-shell' structure: a nucleic acid packaged core and a surrounding envelope protein shell 32,33. The excellent gene delivery ability of viral vectors is due to their sophisticated infection mechanisms. Usually, viruses enter host cells via receptor-mediated endocytosis, sequentially unpacking the envelope and capsid to release the genetic cargo 34. However, undesirable immune responses, limited gene packaging capability or aberrant gene expression limited their clinical applications 35-37. In solving these problems, several reports had revealed that 'core-shell' virus-mimicking gene delivery systems displayed excellently in cancer gene therapy 38-42.

**Figure 1:**
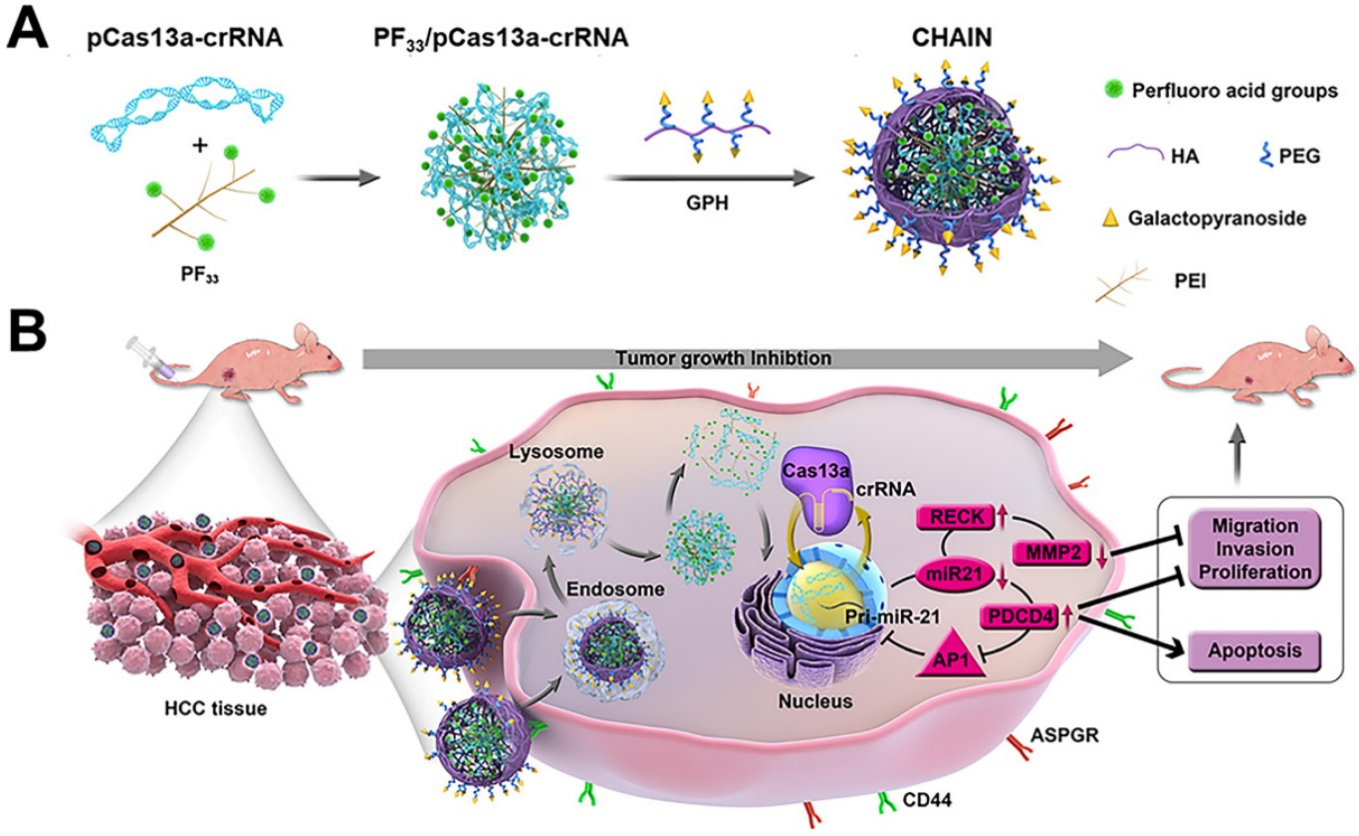
Schematic illustration of preparation and anti-tumor mechanism of hierarchically tumor-activated nanoCRISPR-Cas13a. (**A**) Preparation of CHAIN/pCas13a-crRNA (miR-21). (**B**) CHAIN/pCas13a-crRNA (miR-21) facilitates efficient microRNA disruption for multi-pathway-mediated tumor suppression.

Herein, inspired by the structure and infection pathway of viruses, we designed hierarchically tumor-activated nanoCRISPR-Cas13a (CHAIN) for multi-pathway-mediated tumor suppression by efficient microRNA disruption *in vivo* (Figure 1). A fluorinated polyetherimide (PEI; Mw=1.8KD) with graft rate of 33% (PF_33_) was utilized to compact the pCas13a-crRNA megaplasmid via self-assemble to constitute a nanoscale 'core' (PF_33_/pCas13a-crRNA), which was further wrapped by modified hyaluronan (HA) derivatives (galactopyranoside-PEG_2000_-HA, GPH) to form CHAIN. The CHAIN could long circulate in the blood due to its PEGylated anionic shell, and actively targeting HCC cells via overexpressed asiaglycoprotein receptor (ASPGR) and CD44 receptor with the help of galactopyranoside (Gal) and HA 43,44, respectively. After internalization, HA layer could be decomposed by hyaluronidase (HAase) in the endo/lysosomal system, and the re-exposed cationic inner 'core' which could efficiently promote lysosomal escape and released the pCas13a-crRNA megaplasmid into the cytoplasm. Following the miR-21 downregulation by CRISPR/Cas13a system, reversion‐inducing‐cysteine‐rich protein with Kazal motifs (RECK) and programmed cell death protein 4 (PDCD4) were restored and matrix metalloproteinases-2 (MMP-2) was inhibited. Furthermore, the miR-21-PDCD4-AP-1 positive feedback loop further serves as a reinforcement for cancer therapy. Therefore, CHAIN collectively induced cancer cell apoptosis and minimized cell proliferation, metastasis and invasion. Prospectively, the CHAIN platform offers a promising strategy for multi-signal-pathways regulation in cancer therapy by efficient CRISPR-Cas13a-mediated interference of one oncogenic microRNA.

## Results and discussion

### Preparation and characterization of CHAIN

**Figure 2:**
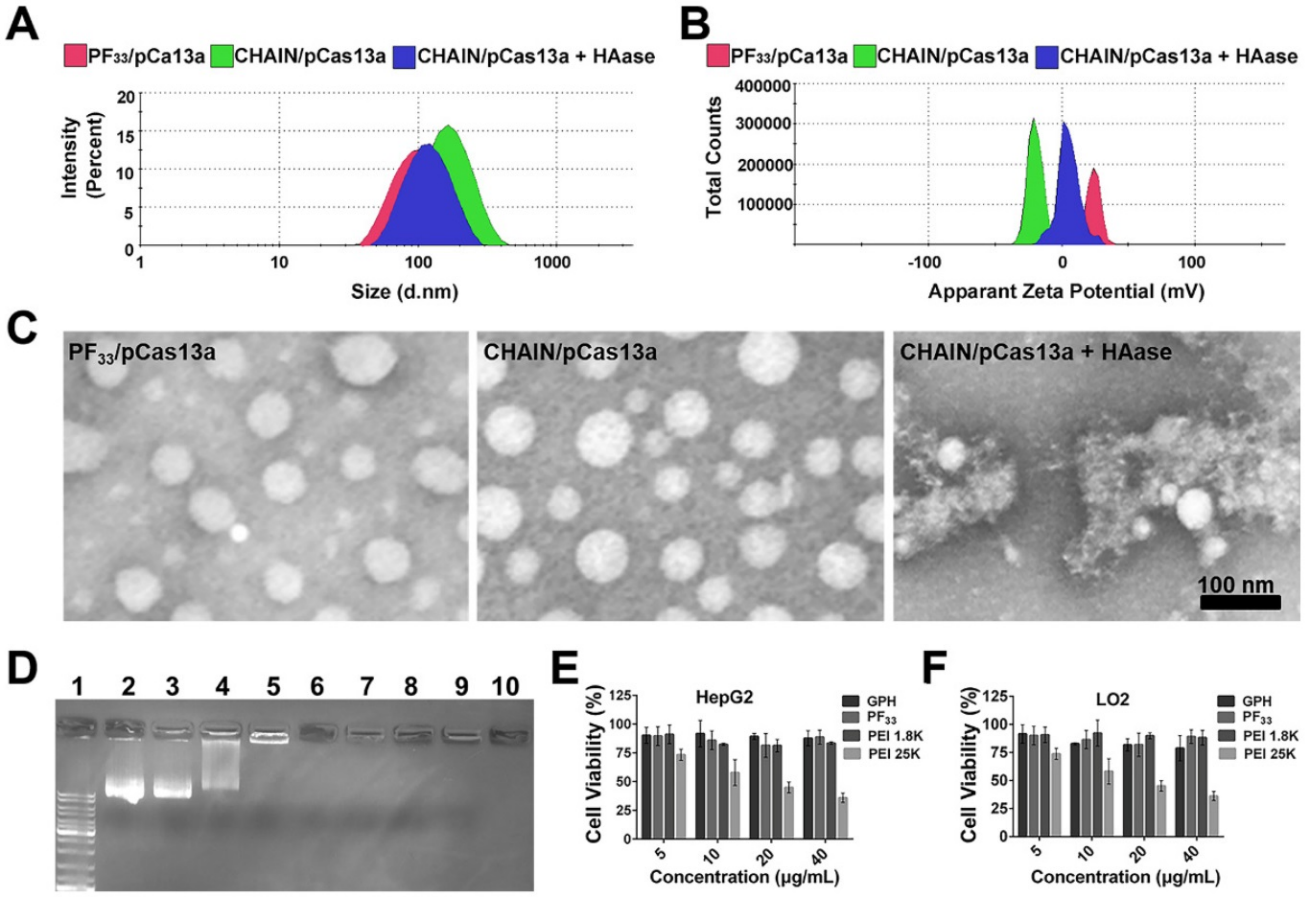
Characterization of CHAIN/pCas13a. Size distribution (**A**), zeta potential (**B**) of PF_33_/pCas13a, CHAIN/pCas13a and HAase-treated CHAIN/pCas13a (CHAIN/pCas13a+HAase). (**C**) Morphologies of PF_33_/pCas13a, CHAIN/pCas13a and HAase-treated CHAIN/pCas13a assessed by TEM. Scale bars, 100 nm. (D) The gel retardation assay of the PF_33_/pCas13a and CHAIN/pCas13a. Lane 1: DNA marker, lane 2: naked pCas13a, lanes 3-9: PF_33_ and pCas13a at mass ratios of 0.125 : 1, 0.25 : 1, 0.5 : 1, 1 : 1, 2 : 1, 5 : 1, and 10 : 1, lane 10, GPH: PF_33_ : pCas13a = 30 : 10 : 1. Cytotoxicity of PF_33_, GPH, PEI 1.8K and PEI 25K in HepG2 (E) and LO2 (F) cells.

PF_33_ (substitution degree of fluorine on PEI is about 33%) and GPH (substitution degree of Gal-PEG on HA is about 33%) were synthesized and then characterized via ^19^*F*-NMR and ^1^*H*-NMR, respectively (Figure S1 and Figure S2). Herein, we first utilized PF_33_ to bind the CRISPR-Cas13 megaplasmid (~ 13 kb). As shown in Figure 2A-B, PF_33_/pCas13a showed a narrow size distribution with an average diameter of 117 ± 4 nm and a positive zeta potential of 23.1 ± 0.3 mV. When PF_33_/pCas13a was coated with the multifunctional polymer (GPH) to form CHAIN/pCas13a, the hydrodynamic diameter shifted to 153 ± 3 nm, while the zeta potential was reduced to -20.8 ± 0.4 mV. Next, the morphology of PF_33_/pCas13a and CHAIN/pCas13a was confirmed by TEM (Figure 2C). Then, we investigated the enzymatic sensitivity of CHAIN/pCas13a. After incubation with HAase, the 'shell' GPH was degraded and dissociated from the PF_33_/pCas13a core (Figure 2C) and the particle size potential of CHAIN/pCas13a decreased from 153 ± 3 nm to 129 ± 5 nm**,** while the zeta potential reversed to 5.4 ± 0.4 mV (Figure 2B). These results suggested that CHAIN was a well-organized 'core-shell' structure and had agile enzyme sensitiveness.

A gel shift assay further confirmed the pCas13a binding ability of PF_33_. When PF_33_ and pCas13a was at mass ratios 1:1, the pCas13a was completely bound by PF_33_, and coating with the negatively charged GPH had no influence on pCas13a encapsulation (Figure 2D). Furthermore, CCK-8 assays were performed to test the cytotoxicity of PF_33_ and GPH on HepG2 and LO2 cells. Compared with PEI 25K, GPH and PF_33_ showed little cytotoxicity on both cell lines (Figure 2E-F).

### Cellular internalization, endo/lysosomal escape, and transfection *in vitro*

The CD44 expression of HepG2 cells was evaluated by flow cytometry before analyzing the cellular internalization efficiency of CHAIN 45. The results showed CD44 was highly expressed (~ 100%) on HepG2 cells (Figure S3). Since ASGPR is an endocytotic receptor expressed primarily on the surface of hepatocytes 46, we did not analyze its expression levels in HepG2 cells.

**Figure 3:**
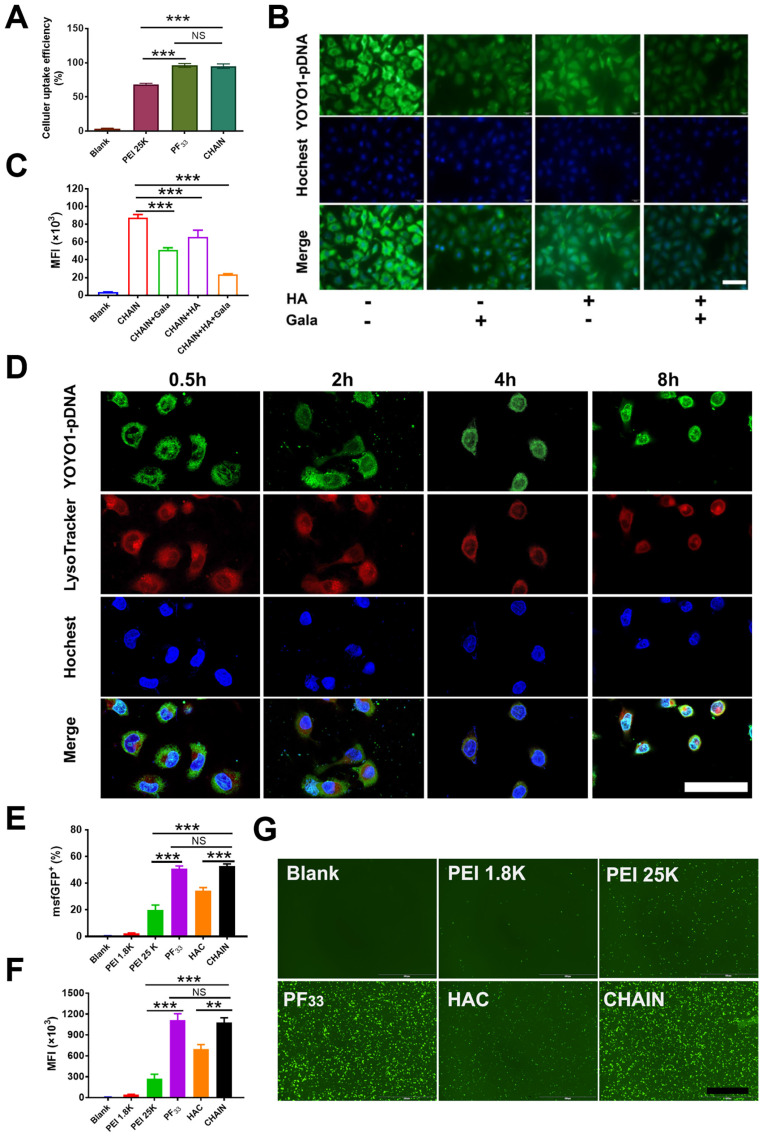
Cellular uptake, endo/lysosomal escape and *in vitro* transfection of CHAIN. (**A**) Quantitative analysis of the cellular uptake efficiency of CHAIN. (**B**) Fluorescence images of cellular uptake in HepG2 cells incubated with CHAIN/pCas13a (with or without free galactopyranoside (1 mM) or/and HA (10 mg/mL) competition). Scale bars, 50 μm. (**C**) Quantitative analysis of MFI. (**D**) The images of intracellular colocalization in HepG2 cells transfeted with CHAIN/pDNA at different time points. YOYO-1 Hoechst and LysoTracker stained separately pDNA, cell nuclei and endo/lysosomes. Scale bars, 20 μm. (**E**) Fow cytometry results of transfection efficiency. (**F**) Quantitative analysis of MFI. (**G**) Fluorescence images of Blank, PEI 1.8K/pCas13a-msfGFP, PEI 25K/pCas13a-msfGFP, PF_33_/pCas13a-msfGFP, HAC/pCas13a-msfGFP and CHAIN/pCas13a-msfGFP taken by inverted fluorescence microscopy. Scale bars: 500 μm.

Then, the cellular uptake efficiency of the YOYO-1-labeled CHAIN/pCas13a was measured in HepG2 cells. As shown in Figure 3A and Figure S4, PF_33_/pCas13a and CHAIN/pCas13a exhibited comparable cellular uptake efficiency (> 95%), which performed more excellent than that of PEI 25K/pCas13a (~ 74%, *p* < 0.001,* p* < 0.01, respectively). Moreover, HAC/pCas13a performed slightly less inferior than CHAIN/pCas13a (Figure S5). In order to verify whether the excellent cellular uptake efficiency of CHAIN/pCas13a was associated with the CD44 receptor and ASGPR, we performed competitive binding experiments. When HepG2 cells were incubated with CHAIN/pCas13a under free galactopyranoside and/or HA, a relatively low mean fluorescence intensity (MFI) was observed, implying that cellular uptake of CHAIN/pCas13a was substantially inhibited (Figure 3B-C and Figure S6). These results indicated that the CHAIN/pCas13a were taken up by HepG2 cells through both ASGPR- and CD44-mediated endocytosis.

In addition to internalization, CHAIN/pCas13a need to escape from the endo/lysosomal system and transfer into the nucleus to initiate the transcription of pCas13a. To evaluate the endosomal escape ability of CHAIN/pCas13a, we performed CLSM to observe the intracellular distribution at different time points. After incubation for 0.5 h, most of the pCas13a (green) was located in the endo/lysosomes (red). As time increased, the pCas13a was gradually transferred from the endo/lysosomes into the nuclei (blue). After 8 h of incubation, most of the pCas13a entered the nuclei of HepG2 cells (Figure 3D), suggesting that CHAIN/pCas13a exhibited excellent endosomal/lysosomal escape ability. Furthermore, the colocalization of red, green and blue fluorescence were analyzed in Figure S7.

Since the transfection efficiency was strongly affected by the size of the plasmid DNA 47, we evaluated the transfection potency of CHAIN and PF_33_ using the Cas13a-msfGFP plasmid (pCas13a-msfGFP, 13.58 kb) in HepG2 cells. PEI 25K was chosen as the control 48,49, which induced ~ 19.1% GFP-positive cells. As shown in Figure 3E-G and Figure S8, PF_33_/pCas13a-msfGFP showed significantly increased transfection efficiency (~ 52.3%, *p* < 0.001), while PEI 1.8K/pCas13a-msfGFP performed poorly (< 3%). Meanwhile, CHAIN/pCas13a-msfGFP also displayed excellent gene transfection potency (~ 54.9%), which was significantly higher than that of HAC/pCas13a-msfGFP (HA-coated PF_33_/pCas13a-msfGFP, used as a control; ~ 37.1%, *p* < 0.01). The improved cellular internalization caused by CD44 and ASGPR receptors-mediated endocytosis as well as the excellent endo/lysosomal escape capacity could be accounted for the excellent gene transfection potency of the CHAIN/pCas13a-msfGFP.

We further investigated whether the serum concentration affected the transfection efficiency. The results showed that of the PF_33_/pCas13a-msfGFP and CHAIN/pCas13a-msfGFP maintained high transfection efficacy (> 50%) in the presence of 5% ~ 30% serum, which was comparable to that of serum-free medium (Figure S9 and Figure S10). These results imply that CHAIN holds great promise for *in vivo* gene delivery.

### Construction of the pCas13a-crRNA expression vectors

**Figure 4:**
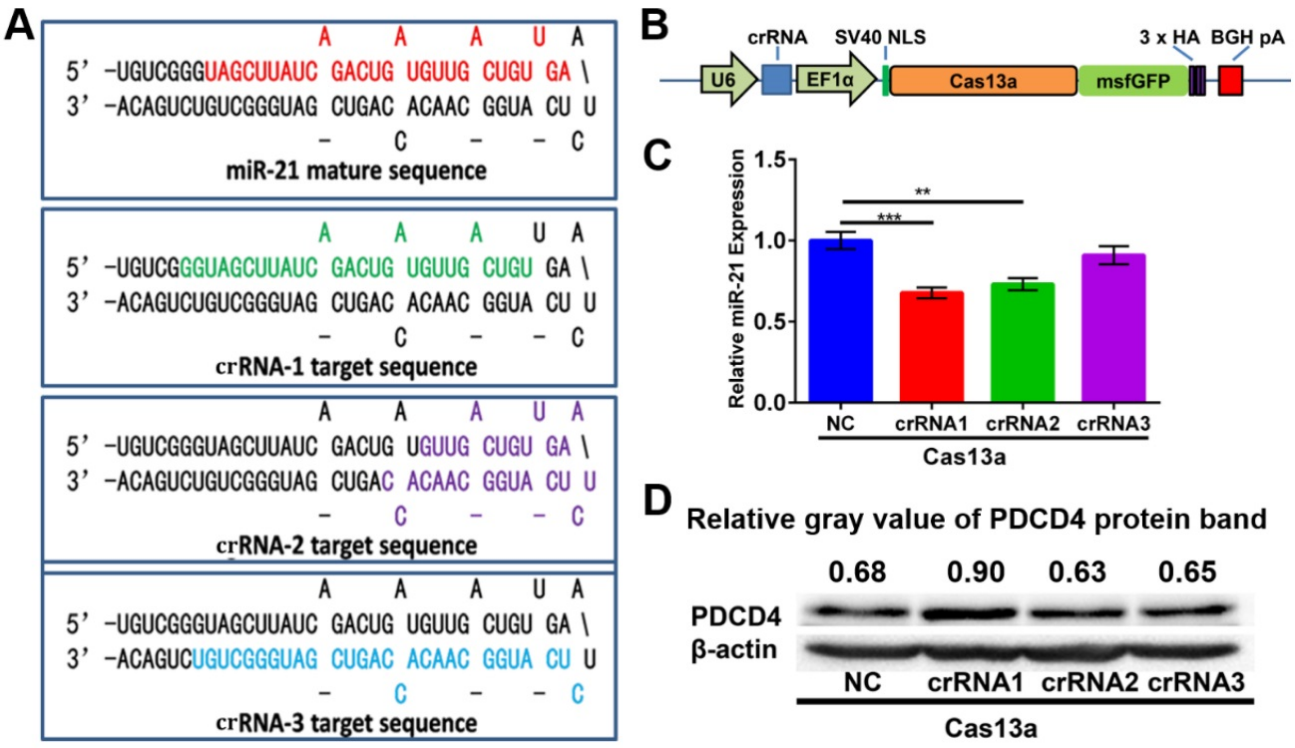
Construction of the 'all-in-one' pCas13a-crRNA expression vectors. (**A**) Design of crRNAs targeting the miR-21 precursor sequences. Mature miR-21 and crRNA sequences in the pre-miR-21 hairpin are highlighted in colors. (**B**) Schematic diagram of the construction of Cas13a and the crRNA 'all-in-one' expression vector. (**C**) MiR-21 expression level examined by stem-loop RT-qPCR. NC, negative control: Cas13a without crRNA, *P*-values are the mean ± SEM (*n* = 3). (** p < 0.01, *** p < 0.001). (**D**) PDCD4 protein expression analyzed via western blotting, NC, negative control: Cas13a without crRNA.

For miR-21 knockdown with the CRISPR-Cas13a system, three individual crRNAs were designed to target primary miR-21. Mature miR-21 sequences and all three crRNA sequences are shown in the precursor miR-21 (pre-miR-21) hairpin structure (Figure 4A). As depicted in Figure 4B, we adopted an 'all-in-one' system, which expresses both the crRNA and Cas13a nuclease driven by the U6 and EF1α promoters, respectively. After introducing CHAIN/pCas13a-crRNA into HepG2 cells by separate transfections, we performed quantitative PCR (qPCR) assays and found that crRNA1 and crRNA2 could induce ~ 37% and ~ 27% reductions in mature miR-21 expression, respectively (Figure 4C), suggesting that this strategy was effective. Moreover, we found that PDCD4, one known miR-21 target genes ^14^, was significantly upregulated, as detected by western blot analysis (Figure 4D). Due to its high efficiency, pCas13a-crRNA1 was chosen for further studies.

### Antitumor analysis *in vitro*

**Figure 5:**
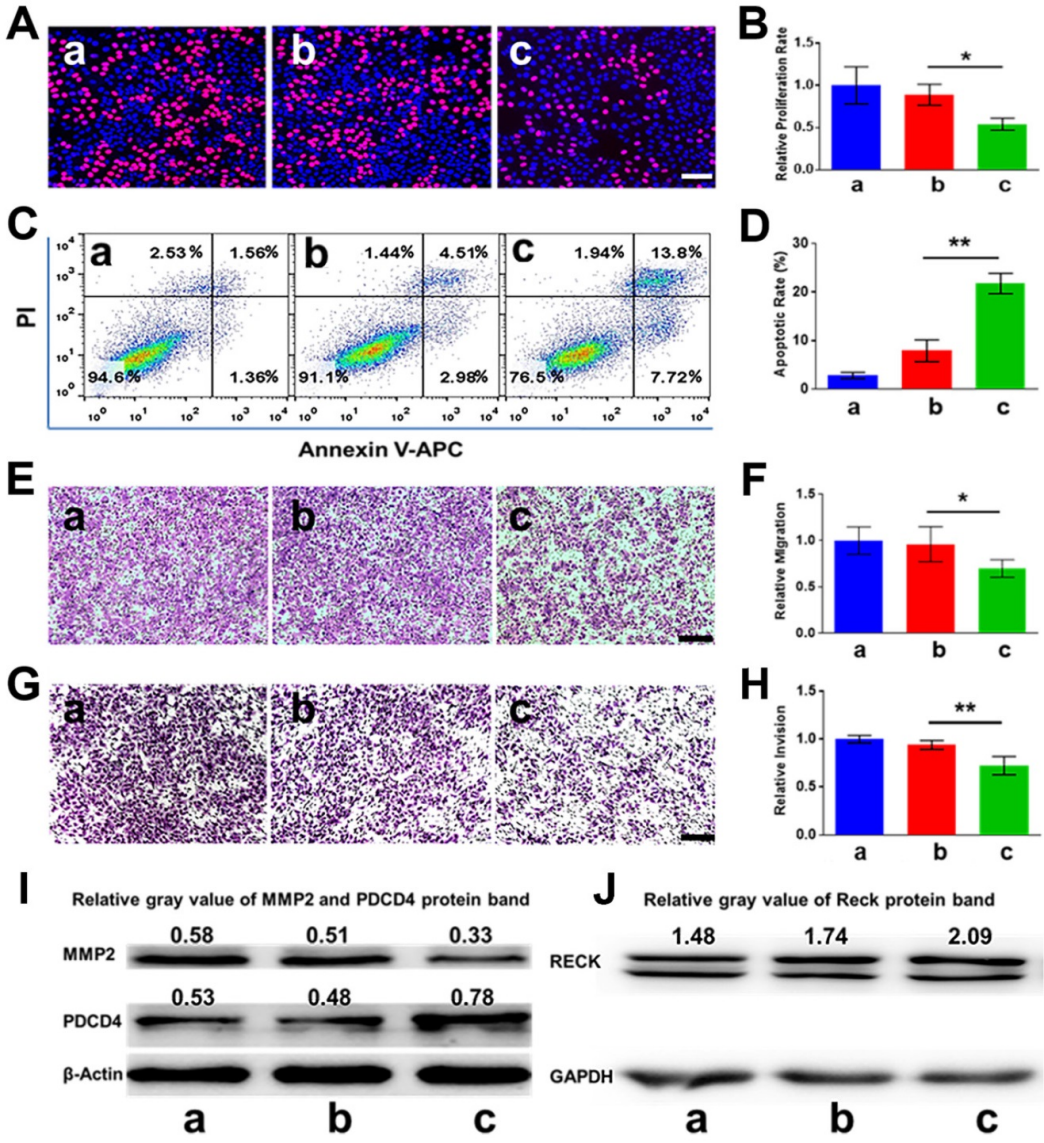
*In vitro* anti-tumor effects of CHAIN. (**A**, **B**) The proliferation of HepG2 cells evaluated via EdU assays (** *p* < 0.01). (**C**, **D**) Apoptosis of HepG2 cells detected by flow cytometry (** p < 0.01). (**E**, **F**) Migration of HepG2 cells treated with CHAIN/pCas13a-crRNA1 or CHAIN/pCas13a (* p < 0.05). (**G**, **H**) Invasion of HepG2 cells treated with CHAIN/pCas13a-crRNA1 or CHAIN/pCas13a (** *p* < 0.01). (**I**) PDCD4 and MMP2 protein levels analyzed by western blotting. (**J**) RECK protein levels analyzed by western blotting. a: blank, b: CHAIN/pCas13a, c: CHAIN/pCas13a-crRNA1.

Since PDCD4 was previously identified as one of the direct target genes of miR-21 in cancer cells, we hypothesized that knockdown of miR-21 may upregulate the expression of PDCD4, thereby inhibiting cell proliferation and inducing apoptosis. As pictured in Figure 5A-B, the cell proliferation rate of the CHAIN/pCas13a-crRNA1 group was ~ 27.1%, remarkably lower than that of the CHAIN/pCas13a (without crRNA) treatment group (~ 44.5%, *p* < 0.05). Moreover, CHAIN/pC13a-crRNA1 treatment led to ~ 20% apoptosis in HepG2 cells, while the apoptotic ratio following CHAIN/pCas13a treatment was only ~ 7% (Figure 5C-D). Western blot analysis revealed that the PDCD4 protein level significantly increased after CHAIN/pCas13a-crRNA1 treatment (Figure 5I).

A number of previous studies have revealed miR-21 was able to promote HCC cells invasion, migration and proliferation. Therefore, we aimed to examine whether RNA knockdown mediated by the CHAIN/pCas13a-crRNA1 abrogated the oncogenic activity of miR-21. To evaluate migration and invasion, we transfected HepG2 cells with the CHAIN/pCas13a-crRNA1 or CHAIN/pCas13a. As shown in Figure 5E-H, compared to the CHAIN/pCas13a-treated or blank group, the CHAIN/pCas13a-crRNA1-treated group showed significantly decreased migration and invasion of HepG2 cells. These results were further confirmed by analysis of MMP2 and RECK protein expression (Figure 5I-J), which was related to cell migration and invasion. The above results indicated CHAIN/pCas13a-crRNA1 may be a promising choice to treat HCC*.*

### Targeting efficacy, biodistribution and antitumor activity *in vivo*

**Figure 6:**
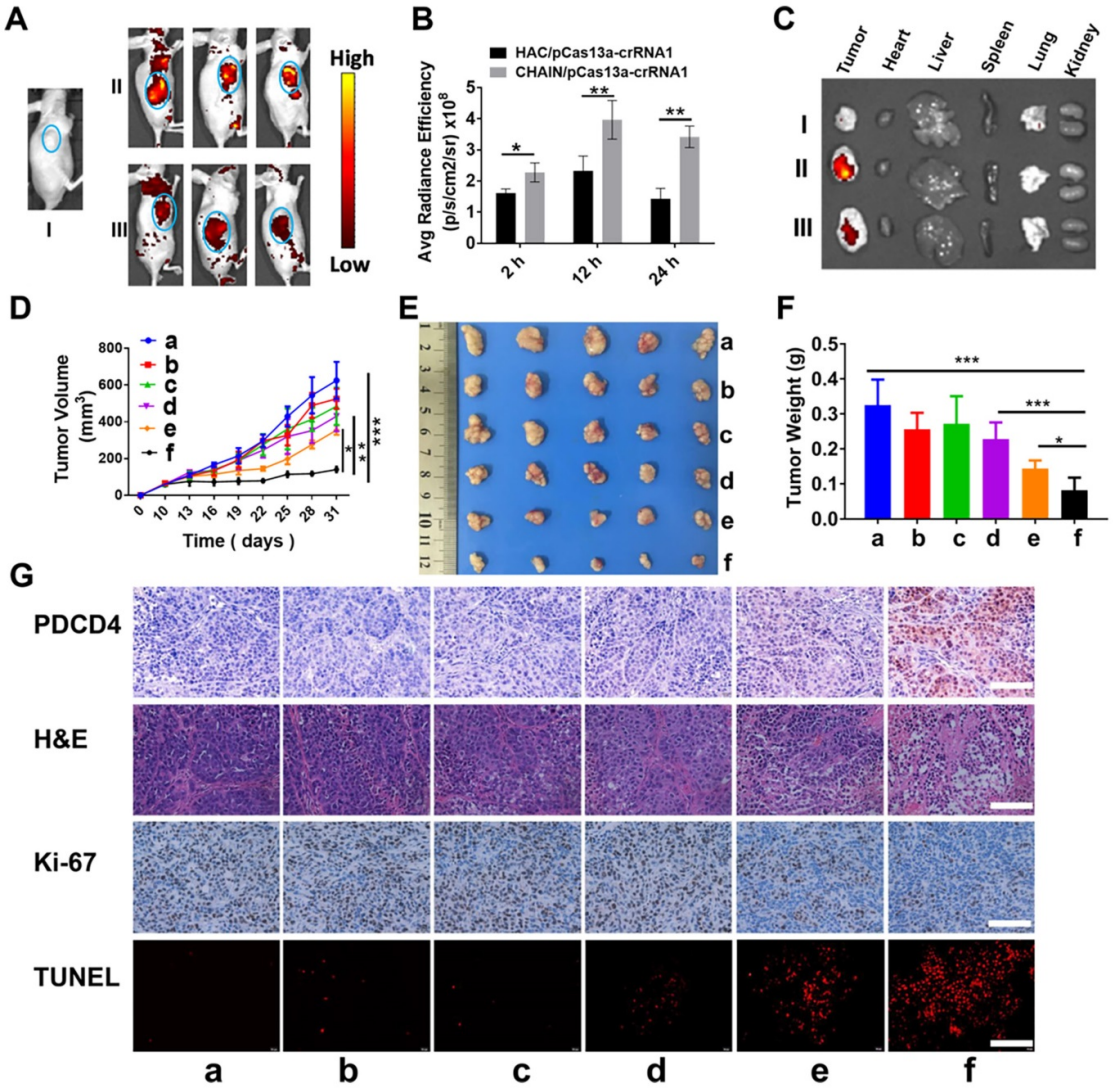
*In vivo* biodistribution and antitumor efficacy of CHAIN. (**A**)* In vivo* fluorescence images of (I) blank, (II) CHAIN/pCas13a-crRNA1 and (III) HAC/pCas13a-crRNA1 groups. (**B**) Quantitative average radiance efficiency of tumors *in vivo*. (**C**) *Ex vivo* images of (I) blank, (II) CHAIN/pCas13a-crRNA1 and (III) HAC/pCas13a-crRNA1 groups. (**D**) Tumor volume in different groups. Values were the mean ± SD (n = 5, * p < 0.05, ** p < 0.01). (**E**) Image of tumors collected from sacrificed nude mice. (**F**) Tumor weights in each treatment group. a: PBS, b: GPH, c: pCas13a-crRNA1, d: CHAIN/pCas13a, e: HAC/pCas13a-crRNA1, f: CHAIN/pCas13a-crRNA1.

To investigate the tumor targeting capability and biodistribution of CHAIN/pCas13a-crRNA1 in subcutaneous xenograft tumor mice, we tracked the fluorescence of CHAIN/pCas13a-crRNA1 (labeled with TOTO-3) at different time points. As described in Figure 6A-B, while both groups rapidly gathered in the tumors at 2 h, the fluorescence intensity was substantially stronger in mice treated with CHAIN/pCas13a-crRNA1 than in mice treated with HAC/pCas13a-crRNA1. The CHAIN/pCas13a-crRNA1 gradually accumulated in the tumor over time. Twenty-four hours after injection, strong fluorescence intensity was still observed at the tumor area of mice injected with CHAIN/pCas13a-crRNA1, while the fluorescence intensity of mice injected with HAC/pCas13a-crRNA1 was weakened. Similarly, stronger fluorescence was observed after CHAIN/pCas13a-crRNA1 treatment than HAC/pCas13a-crRNA1 treatment in *ex vivo* photos (Figure 6C). These results demonstrated that the versatile polymer GPH played an important role in long-term retention and active targeting in HCC tumor tissue *in vivo*.

Encouraged by the excellent inhibitory effect of the CHAIN/pCas13a-crRNA1 on cancer cells *in vitro*, we further investigated the therapeutic effect *in vivo* using HCC xenograft subcutaneous mice model. Compared to those of the groups treated with PBS, GPH, pCas13a-crRNA1 (free plasmid) and CHAIN/pCas13a, the tumor growth of the CHAIN/pCas13a-crRNA1- or HAC/pCas13a-crRNA1-treated group was strongly inhibited (Figure 6D-E). Notably, CHAIN/pCas13a-crRNA1 induced a substantially stronger antitumor effect than HAC/pCas13a-crRNA1 (*p* < 0.05). The antitumor activity of CHAIN/pCas13a-crRNA1 was further validated by the average weight of tumors harvested at the end of the experiment (Figure 6F).

Next, mir-21 expression in the tumors was quantified by qRT-PCR, about 29% decreased in CHAIN/pCas13a-crRNA1-treated group (Figure S11). Moreover, to evaluate the mechanisms that underlie the anti-miR-21 therapy, we performed an immunohistochemical assay. As depicted in Figure 6G, we found that the PDCD4 level was significantly increased after CHAIN/pCas13a-crRNA1 treatment, indicating that miR-21 was dramatically knocked down by the CRISPR-Cas13a system. Then, Ki-67 staining assays and transferase-mediated dUTP nick end labeling (TUNEL) staining assays were used to analyze cell proliferation and apoptosis in the tumors, respectively. The CHAIN/pCas13a-crRNA1 treatment induced a notable reduction in Ki-67-positive cells and a marked augment in TUNEL-positive cells *in vivo*. The above results revealed that anti-miR-21 therapy via the CRISPR-Cas13a system was an effective therapeutic choice.

### Toxicity evaluation *in vivo*

The blood from each mouse was collected for analysis of the complete blood count (CBC) and blood chemistry profile to explore the toxicity of our CHAIN *in vivo*. As shown in Figure S12, the data of all groups demonstrate no significant differences and were comparable to those of normal mice. Meanwhile, histological analysis of the major organs revealed no significant pathological changes (Figure S13). Therefore, the CHAIN/pCas13a-crRNA1 may be a safe therapeutic candidate for applications *in vivo*.

## Materials and methods

### Materials

Heptafluorobutyric anhydride and PEI 1.8K were obtained from Alfa-Aesar. PEI 25K (molecular weight = 25 kDa), N-(3-dimethylaminopropyl)-N'-ethylcarbodiimide hydrochloride (EDCI) and 4-dimethylaminopyridine (DMAP) were purchased from Sigma-Aldrich. 4-aminophenyl β-D-galactopyranoside was provided by Tokyo Chemical Industry Corp., Ltd. (Shanghai, China). NHS-PEG_2000_-OH was synthesized by Zhenzhun Biotechnology Corp., Ltd. (Shanghai, China). Sodium hyaluronate (HA, molecular weight = 35 kDa) was acquired from Freda Biochem Corp., Ltd. (Shandong, China), YOYO-1, TOTO-3, LysoTracker Red, and Hoechst 33342 were obtained from Invitrogen (USA). The CCK-8 Cell Counting Kit was purchased from Vazyme Biotech Corp., Ltd. (Nanjing, China). The apoptosis detection kit was provided by KeyGen Biotech. Corp., Ltd. (Nanjing, China). The EdU detection kit was purchased from Beyotime Biotech. Corp., Ltd. (Shanghai, China). NovoRec®PCR Seamless Cloning Kit was provided by Novoprotein Scientific Inc. (Shanghai, China).

### Synthesis of PF_33_

PF_33_ was synthesized as described previously 50. Briefly, 400 mg PEI 1.8 K and 220 μL heptafluorobutyric anhydride were dissolved separately and mixed in anhydrous methanol. Then, trimethylamine was added to the mixture above and further stirred for 48 h. Eventually, the solution was purified by dialysis with distilled water for 72 h and lyophilized. The final product was verified by ^19^*F* nuclear magnetic resonance (^19^*F* NMR).

### Synthesis of GPH

First, 67.75 mg 4-aminophenyl β-D-galactopyranoside and 106 mg NHS-PEG_2000_-OH were dissolved separately and mixed in N, N-dimethylformamide (DMF). The reaction proceeded under gentle stirring for 6 h at 25 ℃. The product (Gal-PEG_2000_-OH) was purified by dialysis and subsequently lyophilized. 84.6 mg HA, 4.94 mg EDCI and 3.65 mg DMAP were dispersed in 15 mL formamide and stirred for 2 h for activating the carboxyl groups of HA. Then, the mixtures were added to 80 mg Gal-PEG_2000_-OH and stirred for 1 d. Eventually, the solution was dialyzed, lyophilized and the final product was analyzed by ^1^*H* NMR.

### Preparation and characterization of the CHAIN/pCas13a

PF_33_ and pCas13a were incubated with a series of mass ratios for 20 min, subsequently run by electrophoresis (1% agarose gel) for 15 min with 150 V. Afterward, the bands were imaged and recorded by a Gel Doc system (Bio-rad, USA). PF_33_ (20 μg) was mixed with pCas13a (2 μg) by pipetting gently and incubated for 20 min at room temperature. Then, 60 μg pre-dispersed GPH was added and incubated for an additional 25 min to obtain the CHAIN/pCas13a. The nanoparticle sizes and their zeta potentials were measured using a dynamic light scattering (DLS) detector (Zetasizer, Nano-ZS, Malvern, UK). Transmission electron microscopy (TEM, H-600, Hitachi, Japan) was used to observe the morphology of the nanoparticles.

The enzymatic sensitivity of the CHAIN/pCas13a was investigated by incubated with hyaluronidase (HAase) according to the manufacturer's instruction. Then, the nanoparticle characteristics of CHAIN/pCas13a were analyzed as described above.

### Construction of the pCas13a-crRNA expression vector

Three different crRNAs were customized as primer dimers with overhangs and were cloned separately into the BbsI-linearized pC0040-LwaCas13a crRNA backbone plasmid (Addgene, #103851) to generate the crRNA-expressing vectors. For construction of the 'all-in-one' pCas13a-crRNA expression vectors, U6-crRNA was amplified by PCR from pC0040-LwaCas13a crRNA-expressing vectors, followed by ligation to the SpeI- and PacI-digested plasmid pC014-Cas13a-msfGFP (Addgene, #91902) using the NovoRec®PCR Seamless Cloning Kit according to the instructions. All vectors were further analyzed by Sanger sequencing. The U6-crRNA primers sequence were: F: 5'-TGACATTGATTATTGACTAGTGAGGGCCTATTTCCCATGA-3' and R: 5'-TTATCCATCTTTGCATTAATTAACAAAAAATTGTCTTCGTCCCAG-3'.

### Cell lines and cell culture

HepG2 (human HCC cell line) and LO2 (human nontumor hepatic cell line) were provided by the American Type Culture Collection (ATCC, MD). The cells were cultured in Dulbecco's modified Eagle's medium (Gibco, USA), which contained 10% fetal bovine serum (FBS) and 100 units/mL penicillin/streptomycin antibiotics. All cells were cultured in humidified condition at 37 °C with 5% CO_2_.

### Cell viability assay

CCK-8 assay was used to analyze the cytotoxicity of PF_33_ and GPH. LO2 and HepG2 cells were seeded into 96-well plates with incubation for 16 h. Subsequently, PF_33_, GPH, PEI 1.8K, and PEI 25K were administered at concentrations ranging from 0 μg/mL to 40 μg/mL. Two days later, CCK-8 was added and further incubated for 2 h. Finally, the absorbance at 450 nm of each well was recorded *via* a microplate reader (Bio-Rad 680, USA).

### Cellular uptake

The CD44 expression of HepG2 cells were verified with APC anti-mouse CD44 (Biolegend, USA) antibody staining and analyzed by a flow cytometer (ACEA NovoCyte, USA).

For cellular uptake analysis, HepG2 cells (1 × 10^5^ cells per well) were seeded into 12-well plates and incubated overnight. Next, pCas13a was stained with the nucleic acids probe YOYO-1. CHAIN loaded with 1 μg of pCas13a were incubated with the cells for 2 h. For the competitive assay, HepG2 cells were preincubated with free HA (10 mg/mL) and/or with galactopyranoside (1 mM) for 2 h to block the CD44 and/or ASGPR receptors. Then, cells were either analyzed by flow cytometry or washed, fixed and observed by a fluorescence microscope (Olympus, Japan).

### Intracellular trafficking

The intracellular colocalization of CHAIN/pCas13a in the HepG2 cells were performed using confocal laser scanning microscopy (Zeiss, LSM 880, Germany). Cells were seeded into a 12-well plate pre-covered with glass cover slips (1 × 10^5^ cells/well) for 24 h incubation. Next, the CHAIN/pCas13a containing 1 μg YOYO-1-labeled plasmid was added to each well. The cells were incubated for 0.5, 1, 4 or 8 h, rinsed with PBS, stained with LysoTracker Red probe, fixed with 4% paraformaldehyde, and stained with Hoechst in sequence. At last, the cells were subjected to confocal laser scanning microscopy for observation.

### Transfection efficiency

HepG2 cells (1 × 10^5^ cells/well) were seeded into 12-well plates and incubated overnight. Next, the medium was replenished with 500 µL per well fresh medium supplemented with 0 ~ 30% serum. CHAIN encapsulating 1 μg pCas13a-msfGFP were incubated with cells for 6 ~ 8 h, then the medium was discarded and replenished with complete medium and further incubated for 48 h. Meanwhile, PEI 1.8K and PEI 25K were exploited as controls. Finally, GFP expression was imaged and analyzed by flow cytometry.

### RT-qPCR

For detection of mature miR-21 expression, we used a previously described method 51,52, the primers for U6 and miR-21 were synthesized by Tsingke Biotech Corp., Ltd (Chengdu, China). In briefly, total RNA extracted by Trizol Reagent (Ambion, USA) was analyzed by a NanoDrop 2000 system (Thermo Scientific, USA). Reverse transcription assays for miR-21 and U6 small RNA were carried out using a PrimeScript RT reagent Kit (Takara, Japan). Then, qPCR was conducted using SYBR Premix Ex Taq II (Takara, Japan) on a Real-Time PCR system (Bio-Rad, USA). The DNA sequence of U6 and miR-21 primers have been added to the method. The primers for miR-21 were as follows: stem-loop RT primer: 5'-GTCGTATCCAGTGCAGGGTCCGAGGTATTCGCACTGGATACGACTCAACA-3'; forward 5'-GCCCGCTAGCTTATCAGACTGATG-3' and reverse 5'-GTGCAGGGTCCGAGGT-3'. The primers for U6 were as follows: RT primer: 5'-GTGCAGGGTCCGAGGT-3'; forward 5'-GCGCGTCGTGAAGCGTTC-3' and reverse 5'-GTGCAGGGTCCGAGGT-3'. The relative expression of miR-21 was evaluated using U6 small RNA as an endogenous control.

### Western blotting

Western blotting analysis was performed according to the previously described method 53. Briefly, cells were lysed using RIPA reagent (Thermo, USA) on ice for 0.5 - 1 h, and centrifuged at 12,000 rpm for 10 min. Lastly, supernatants were acquired and quantified using a BCA protein assay kit (Thermo, USA), and then, 35 µg protein sample was loaded into each well and subsequently run by SDS-PAGE gels for protein separation and then transferred to 0.45 µm PVDF membranes (Millipore, Germany) for immunoblotting. The PVDF membranes were incubated with 5% nonfat milk and then stained with primary antibodies against β-actin, GAPDH, PDCD4, MMP2 and RECK (Santa Cruz, USA) at room temperature for 2 h. Additionally, the membranes were washed and hybridized with a HRP-conjugated secondary antibodies (Santa Cruz, USA). A chemiluminescence detection system (Clinx, Shanghai) was used to detect the targeted bands.

### *In vitro* migration and invasion assays

HepG2 cells migration and invasion were analyzed using transwell chambers (Millipore, Germany). For the detection of tumor cell migration, after transfection for 48 h, the CHAIN/pCas13a-crRNA1 and CHAIN/pCas13a-transfected cells were trypsinized and suspended, and then, HepG2 cells (50000 cells/well) in serum-free medium were seeded into the upper chamber. The lower chamber of each well was loaded with complete medium. For the detection of tumor cell invasion, matrigel (BD, USA) was used to precoat transwell chambers. Cells were added in serum-free medium, then the complete medium in the lower chamber was served as a chemoattractant. One day or two days later, cells were treated with cooled ethanol, followed by 0.1% crystal violet staining and counted via a microscope.

### Proliferation and apoptosis analysis

For analysis of proliferation and apoptosis, HepG2 cells were incubated in 12-well plates for 16 h, and then transfected by CHAIN/pCas13a-crRNA1 or CHAIN/pCas13a in serum-free medium. Six hours later, the medium was discarded and replenished with complete medium and maintained for another 2 d. Proliferation analysis and apoptosis assays were performed with an EdU detection kit and Annexin V-APC/PI Apoptosis Detection Kit.

### *In vivo* biodistribution

6-week-old female Balb/c nude mice were kept in a specific pathogen-free condition. The animal care and experiment conductions were in accordance with the relevant protocol, which was approved by the Institutional Animal Care and Treatment Committee of Sichuan University (Chengdu, China).

For construction of the HepG2-bearing tumor model, the right flank of each mouse was subcutaneously injected with 1 × 10^7^ cells. Then, mice were randomly divided into 3 groups until the tumor size reached ~ 200 mm^3^, and then intravenously injected with PBS, CHAIN/pCas13a-crRNA1 or HAC/pCas13a-crRNA1 (pCas13a-crRNA1 labeled with TOTO-3). At 2 h, 12 h and 24 h, imaging data were collected utilizing an IVIS Lumina imaging system (Caliper, USA). 24 h later, mice were sacrificed, and tumors and major organs were harvested and imaged.

### *In vivo* antitumor effect

The HepG2 xenograft tumor model was established in accordance with the method described above. 10 days later, mice were divided randomly into 6 groups (5 mice per group). PBS, GPH, pCas13a-crRNA1, CHAIN/pCas13a, HAC/pCas13a-crRNA1, and CHAIN/pCas13a-crRNA1 were prepared freshly and intravenously injected at an interval of 2 days. The individual tumor volumes were measured using a digital caliper every 3 days. 31 days later, the mice were sacrificed, and then the blood was collected for routine blood test and blood chemistry profile. The tumors were collected, weighed and then fixed with 4% paraformaldehyde. Then hematoxylin and eosin (H&E) and immunohistochemical analysis were conducted. Additionally, the major organs were fixed for H&E analysis.

### Statistical analysis

Quantitative data were expressed as standard error of the mean or mean ± standard deviation. *P*-values analysis between groups was calculated using one-way ANOVA method. Significant differences are suggested by NS (not significantly different), * (*p* < 0.05), ** (*p* < 0.01) and *** (*p* < 0.001).

## Conclusion

In summary, inspired by the structure and infection pathway of viruses, we constructed a versatile 'core-shell'-shaped CHAIN for multi-pathway-mediated tumor suppression by efficient delivery CRISPR-Cas13a megaplasmid system *in vivo*. The CHAIN enhanced the cellular uptake by the dual-targeting effect, promoted endo/lysosomal escape, and thus achieving high transfection efficiency of CRISPR-Cas13a megaplasmid in HCC cells. Furthermore, the versatile polymer GPH endowed the CHAIN with stabilization in physiological conditions, long-lasting circulation in the blood and a tumor active targeting capability in subcutaneous HCC-bearing mouse models. Finally, when the CRISPR-Cas13a system was delivered by the CHAIN *in vivo*, it knocked down the targeted oncogene miR-21, restored PDCD4 and RECK, crippled downstream MMP-2, and eventually suppressed tumor growth. Therefore, our CHAIN provides a high-efficient system for CRISPR-Cas13a megaplasmid transfection *in vivo* and achieves multi-signal-pathways regulation by efficient interference of one oncogenic microRNA.

While our work successfully demonstrated the use of CRISPR-Cas13a to target miRNA-21 and regulate RECK, PDCD4, and MMP2, it is important to note that miRNA-21 plays critical roles in other signaling pathways that warrant further research. In addition, it is crucial to investigate the potential impact of unwanted nanoparticle accumulation in other organs on these pathways. Finally, we aim to improve the delivery efficiency of CHAIN to further enhance its efficacy.

## Supplementary Material

Supplementary figures.
