# Supplementary material for: Hierarchically tumor-activated nanoCRISPR-Cas13a facilitates efficient microRNA disruption for multi-pathway-mediated tumor suppression
Source: Theranostics. 2023 May 8;13(9):2774–86. doi: 10.7150/thno.81776 (PMC10240819; doi:10.7150/thno.81776)
Supplement: Supplementary file 1 — Supplementary figures. [file thnov13p2774s1.pdf]

Supplementary Material

Hierarchically tumor-activated nanoCRISPR-Cas13a facilitates efficient microRNA disruption for multi-pathway-mediated tumor suppression

Xiaowei Liu [a, †], Suleixin Yang [a, †], Li Wang [a, †], Xinyue Wu [a], Xinxin Wang [a], Chunqing Ou [a], Jin Yang [a], Linjiang Song [b], Shiyao Zhou [a], Qinjie Wu [a,*], Changyang Gong [a,*]

[a] State Key Laboratory of Biotherapy and Cancer Center, West China Hospital, Sichuan University, Chengdu 610041, P. R. China

[b] School of Medical and Life Sciences, Chengdu University of Traditional Chinese Medicine, Chengdu, 611137, P. R. China

* Corresponding author at: State Key Laboratory of Biotherapy and Cancer Center, West China Hospital, Sichuan University, Chengdu 610041, P. R. China.

E-mail addresses: chygong14@163.com (C Gong) and cellwqj@163.com (Q Wu).

[†] These authors contributed equally to this work.

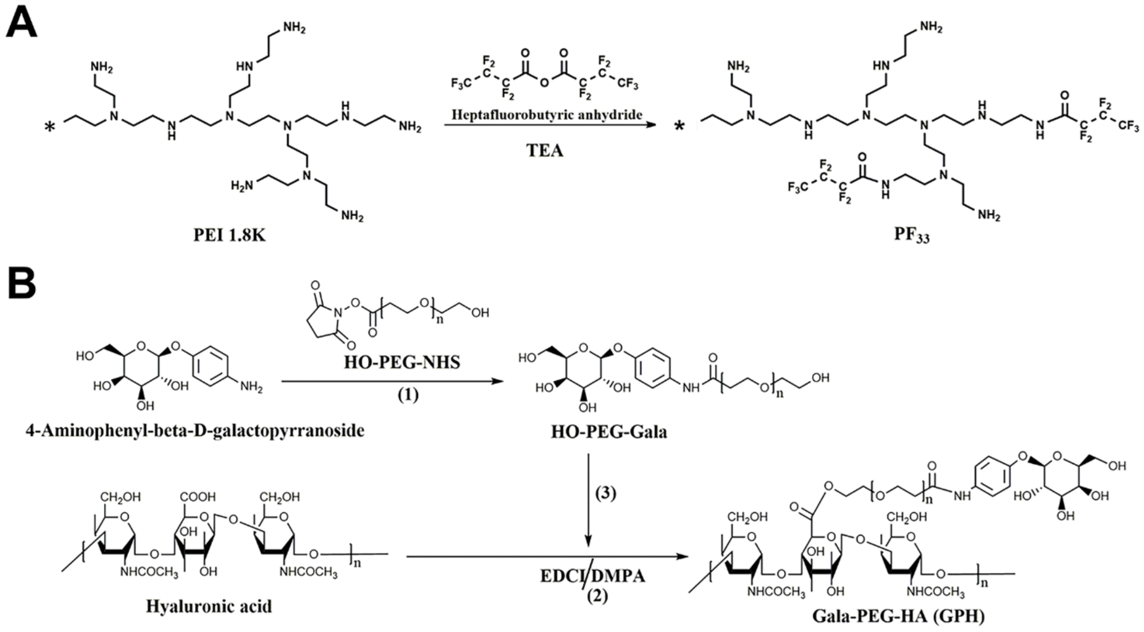

Figure S1. Synthetic procedure of PF_{33} (**A**) and GPH (**B**).

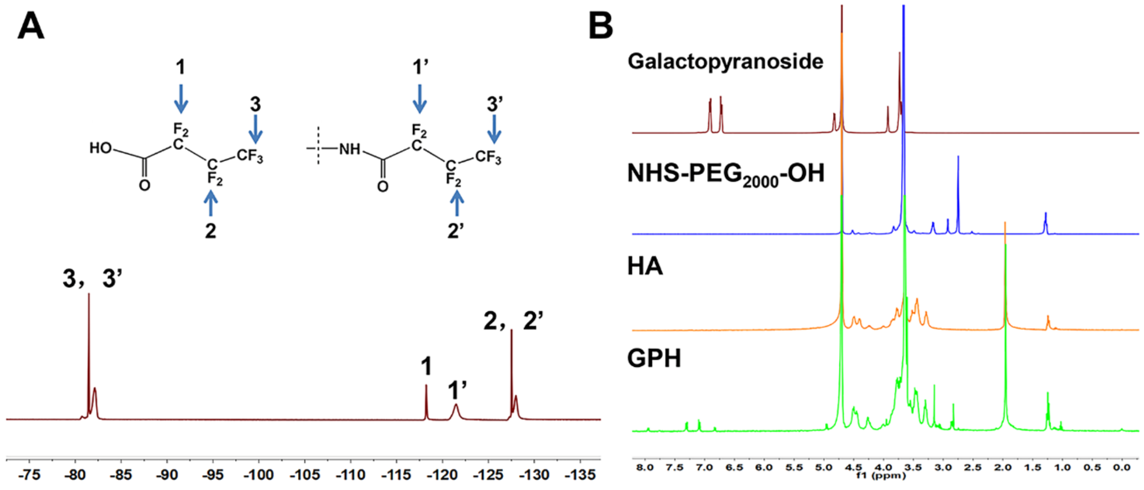

Figure S2. Characterization of PF_{33} and GPH. (**A**) The ^{19}F-NMR spectra of PF_{33}; (**B**) The ^{1}H-NMR spectra of GPH and raw materials.

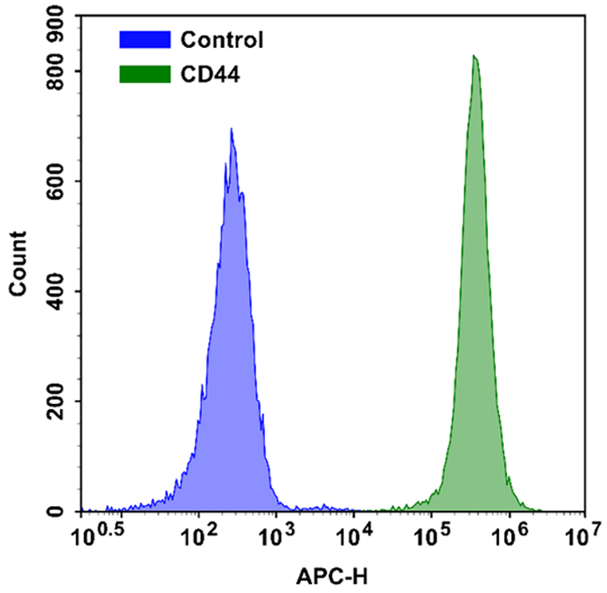

Figure S3. Analysis of the CD44 receptor expression on HepG2 cells by flow cytometry.

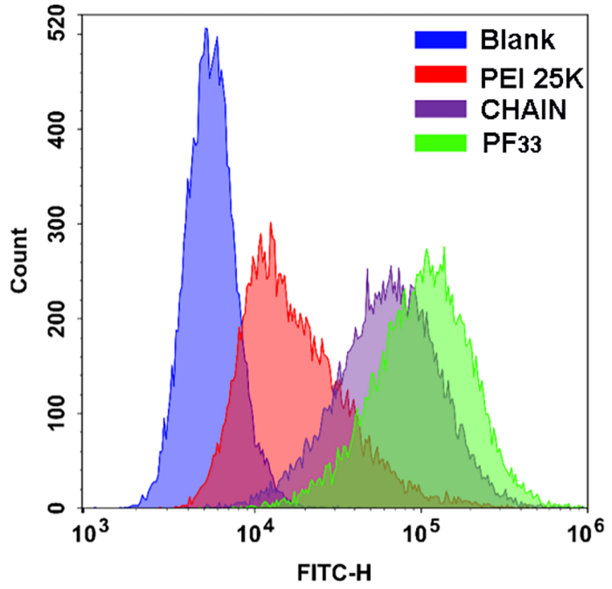

Figure S4. Cellular uptake of HepG2 cells incubated with CHAIN/pCas13a determined by flow cytometry.

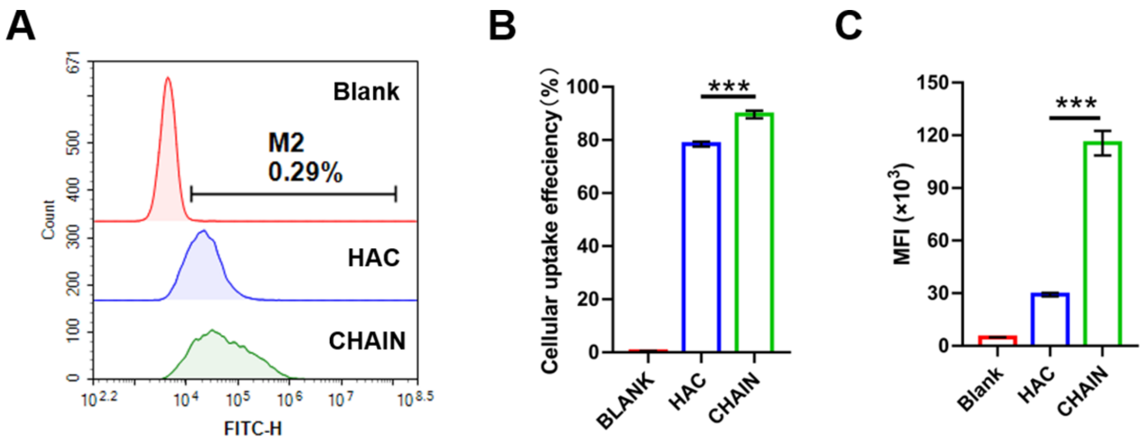

Figure S5. Cellular targeting of HepG2 cells incubated with CHAIN/pCas13a and HAC/pCas13a determined by flow cytometry. (**A**) Flow cytomery data; (**B**) Uptake efficiency (*** $p < 0.001$); (**C**) Mean fluoresence intensity (*** $p < 0.001$).

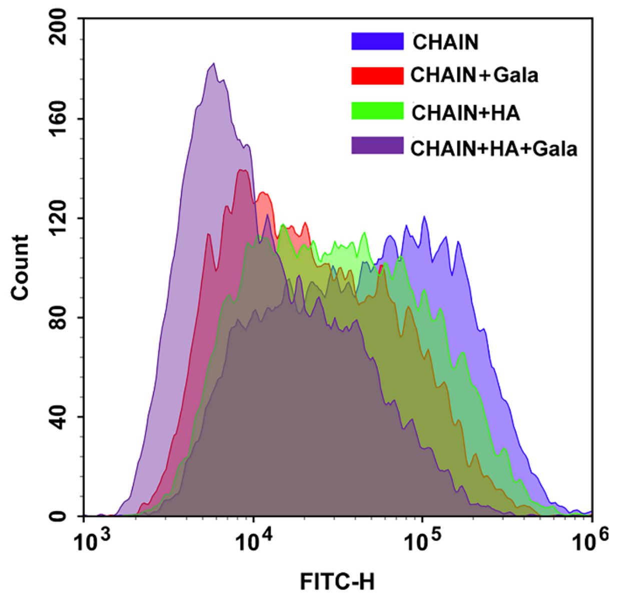

Figure S6. Cellular uptake in HepG2 cells incubated with CHAIN/pCas13a (with or without free galactopyranoside (1 mM) or/and HA (10 mg/mL) competition) determined by flow cytometry.

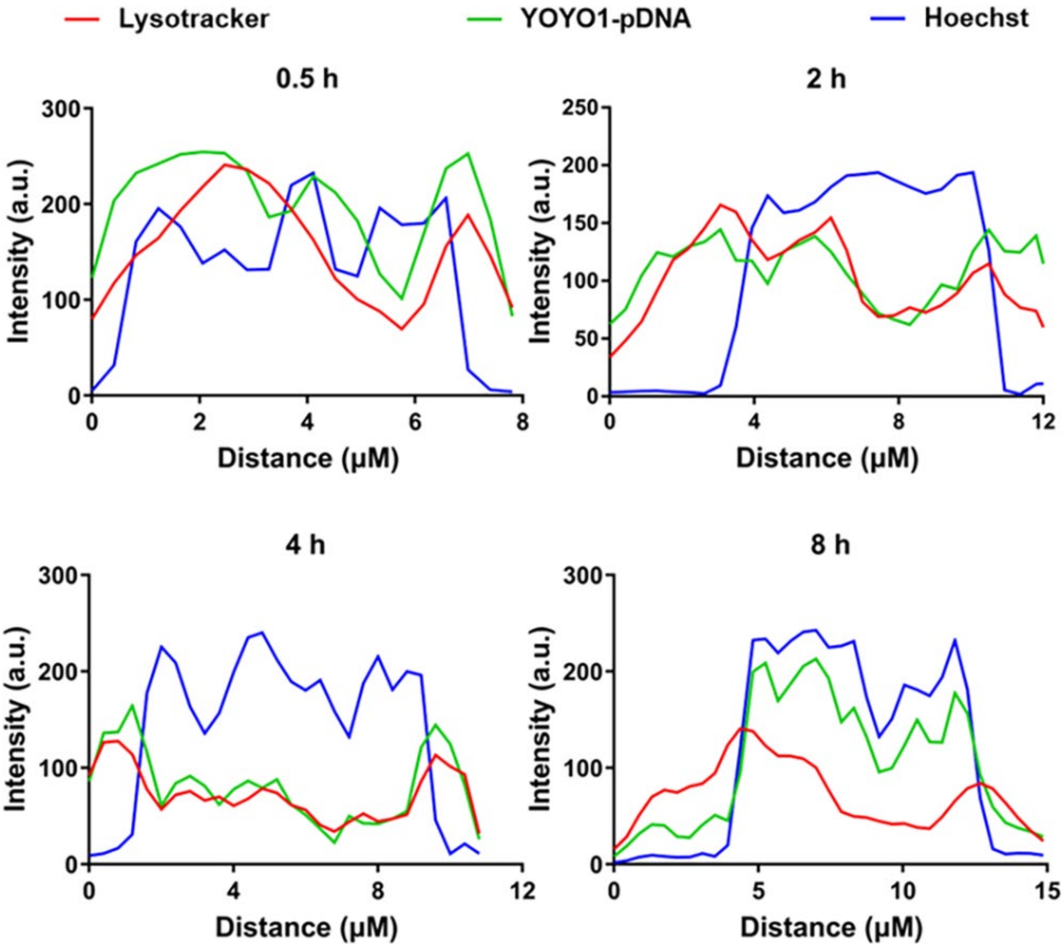

Figure S7. Colocalization of red (Lysotracker), green (YOYO-1-pDNA) and blue (Hoechst) fluorescence of merged cells from Figure 3D.

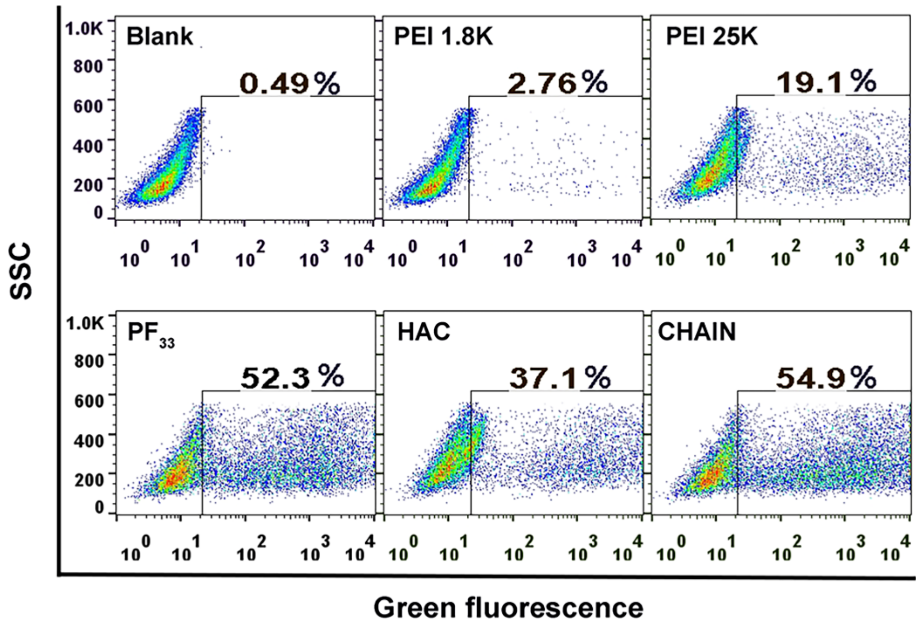

Figure S8. Transfection efficiency of different groups determined by flow cytometry.

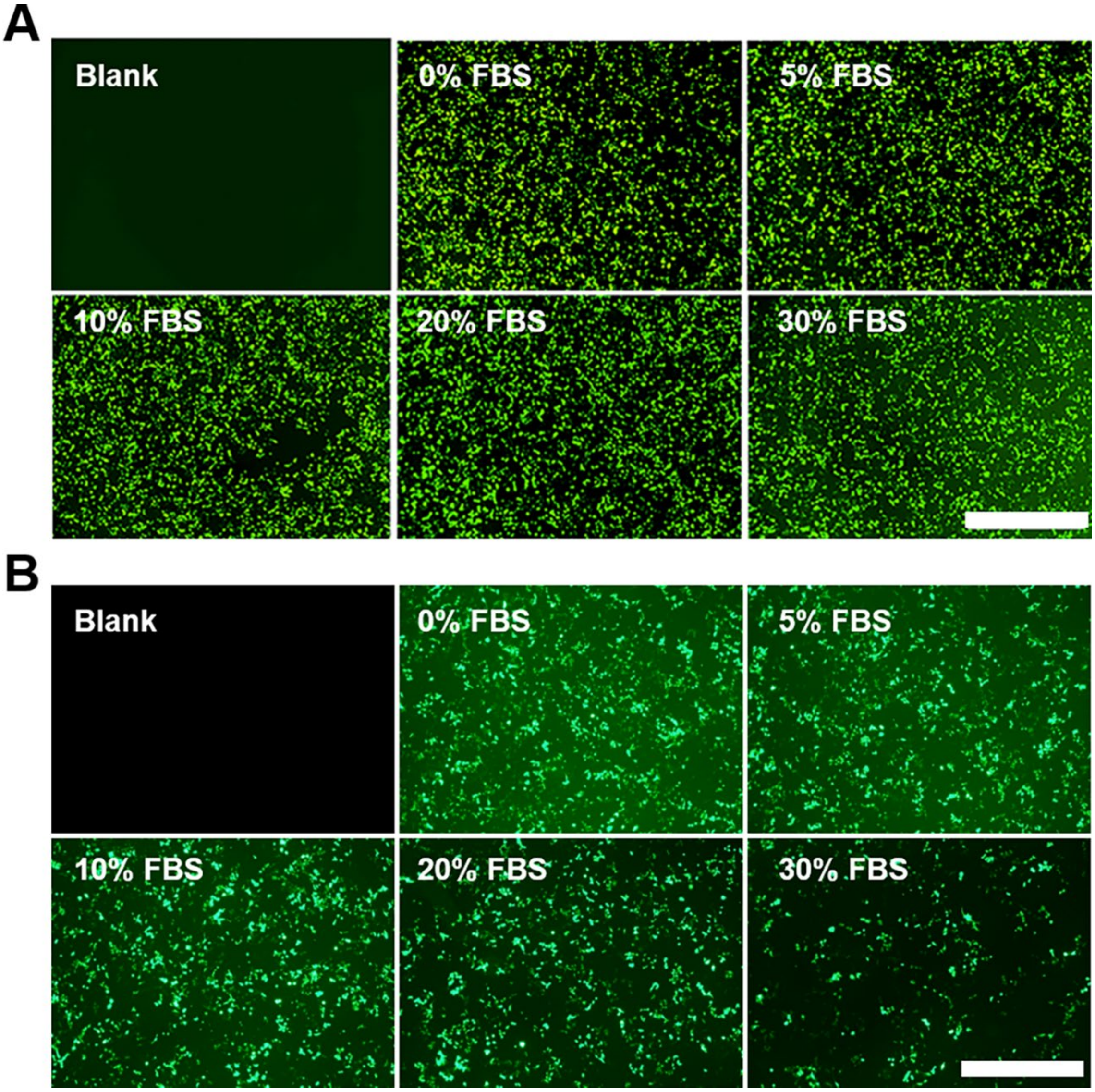

Figure S9. Fluorescence images of CHAIN/pCas13a-msfGFP (**A**) and PF_{33}/pCas13a-msfGFP (**B**) in medium containing 0~30% serum in HepG2 cells. Scale bars: 500 μm.

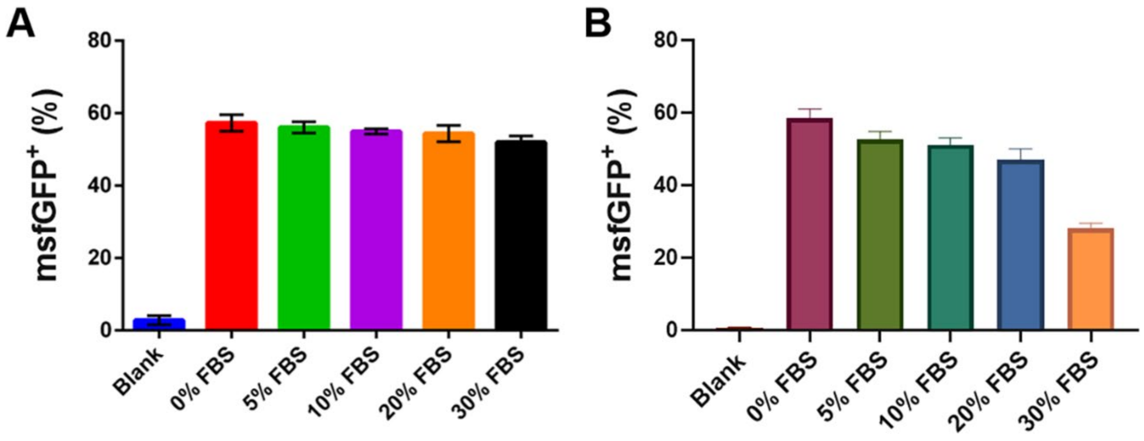

Figure S10. Analysis of the transfection efficiency of CHAIN/pCas13a-msfGFP (**A**) and PF33/pCas13a-msfGFP (**B**) in medium containing 0~30% serum in HepG2 cells.

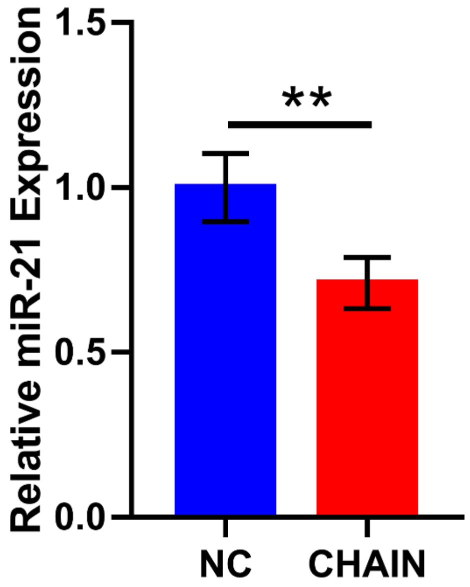

Figure S11. *In vivo* miR-21 expression examined by stem-loop RT-qPCR. P-values are the mean ± SEM (n = 5, ** $p < 0.01$).

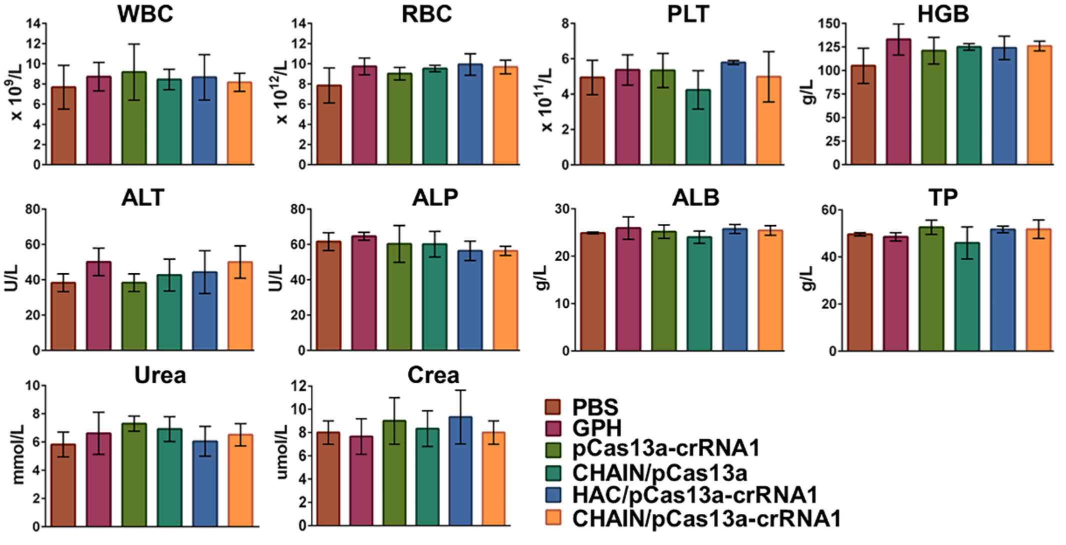

Figure S12. CBC test and blood chemistry profile analysis after different treatments.

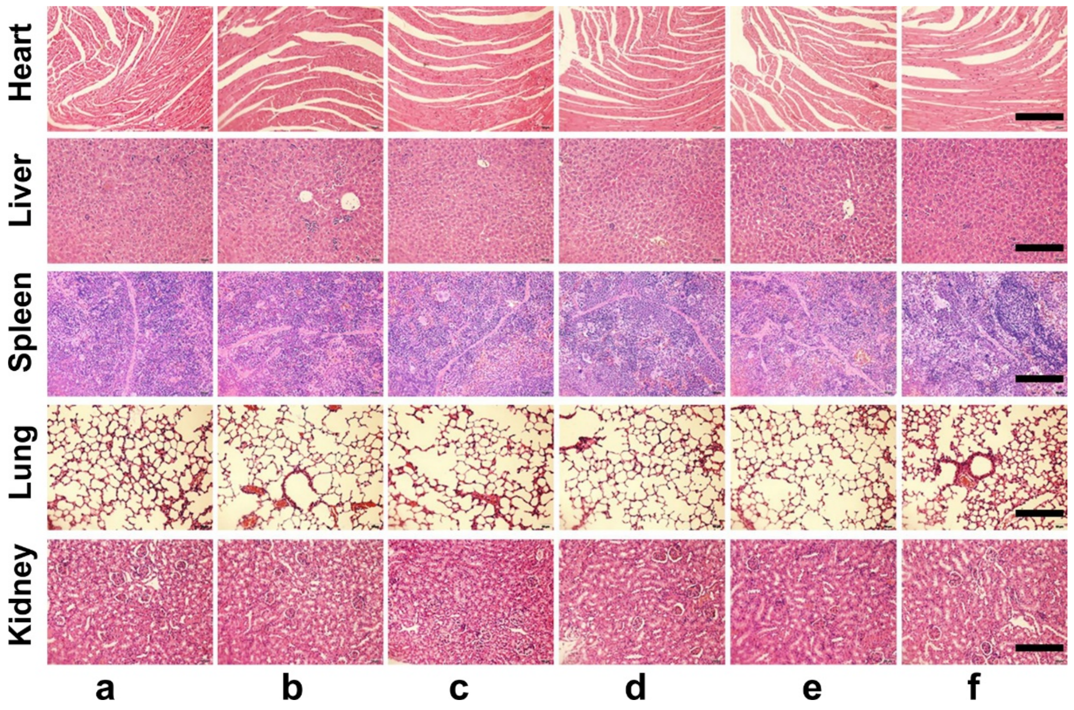

Figure S13. Representative histological sections of major organs stained with H&E. a: PBS, b: GPH, c: pCas13a-crRNA1, d: CHAIN/pCas13a, e: HAC/pCas13a-crRNA1, and f: CHAIN/pCas13a-crRNA1. Scale bars, 20 μm.
